# Pathophysiology of SARS-CoV-2 in Lung of Diabetic Patients

**DOI:** 10.3389/fphys.2020.587013

**Published:** 2020-12-10

**Authors:** Tales Lyra Oliveira, Igor Santana Melo, Léia Cardoso-Sousa, Igor Andrade Santos, Mohamad Bassim El Zoghbi, Caroline Gusson Shimoura, Renata Pereira Georjutti, Olagide Wagner Castro, Luiz Ricardo Goulart, Ana Carolina Gomes Jardim, Thúlio Marquez Cunha, Robinson Sabino-Silva

**Affiliations:** ^1^Heart Institute, Faculty of Medicine, University of São Paulo, São Paulo, Brazil; ^2^Medical School, Municipal University of São Caetano do Sul, São Paulo, Brazil; ^3^Institute of Biological Sciences and Health, Federal University of Alagoas, Alagoas, Brazil; ^4^Department of Physiology, Institute of Biomedical Sciences, Federal University of Uberlândia, Uberlândia, Brazil; ^5^Laboratory of Virology, Institute of Biomedical Sciences, Federal University of Uberlândia, Uberlândia, Brazil; ^6^Department of Physiology and Anatomy, University of North Texas Health Science Center, Fort Worth, TX, United States; ^7^Institute of Biotechnology, Federal University of Uberlândia, Uberlândia, Brazil; ^8^Department of Medical Microbiology and Immunology, University of California, Davis, Davis, CA, United States; ^9^Department of Pulmonology, School of Medicine, Federal University of Uberlândia, Uberlândia, Brazil

**Keywords:** betacoronavirus infection, angiotensin-converting enzyme, SGLT1, diabetes mellitus, pneumonia

## Abstract

Novel coronavirus disease (COVID-19) is an infectious disease caused by severe acute respiratory syndrome coronavirus 2 (SARS-CoV-2). Its impact on patients with comorbidities is clearly related to fatality cases, and diabetes has been linked to one of the most important causes of severity and mortality in SARS-CoV-2 infected patients. Substantial research progress has been made on COVID-19 therapeutics; however, effective treatments remain unsatisfactory. This unmet clinical need is robustly associated with the complexity of pathophysiological mechanisms described for COVID-19. Several key lung pathophysiological mechanisms promoted by SARS-CoV-2 have driven the response in normoglycemic and hyperglycemic subjects. There is sufficient evidence that glucose metabolism pathways in the lung are closely tied to bacterial proliferation, inflammation, oxidative stress, and pro-thrombotic responses, which lead to severe clinical outcomes. It is also likely that SARS-CoV-2 proliferation is affected by glucose metabolism of type I and type II cells. This review summarizes the current understanding of pathophysiology of SARS-CoV-2 in the lung of diabetic patients and highlights the changes in clinical outcomes of COVID-19 in normoglycemic and hyperglycemic conditions.

## Physiological Reviews Summary

(1) The airway surface liquid (ASL) plays a pivotal role in lung defense. Diabetes is related with higher ASL glucose concentration, ASL volume accumulation in alveolar space, imbalance of reactive oxidative species (ROS), and inflammatory chemokine production.
(2) The COVID-19 infection promotes injuries in type I and type II pneumocytes and lung endothelial lesions, with subsequent additional secretion of protein-rich exudate in the alveolar space and intravascular coagulation in lung vessel, which leads to a reduction in surfactant and gas exchange.
(3) The association between diabetes and SARS-CoV-2 increases the glucose and protein concentration in ASL, leading to increase the risk of pneumonia.

(4) The prevalence and severity of hypoxemia and severe hyperinflammation is higher in COVID-19 diabetic patients.
(5) The harmful clinical outcomes and mortality rate of COVID-19 are higher in diabetic subjects.

## Background

### COVID-19 Scenario

Currently, there are more than 38 million infected cases and more than 1,000,000 deaths confirmed worldwide due to the spread of the severe acute respiratory syndrome coronavirus 2 (SARS-CoV-2), the pathological agent of coronavirus disease 2019 (COVID-19) (WHO, 2020). Transmission of SARS-CoV-2 occurs through respiratory droplets exhaled during coughing and sneezing by symptomatic and asymptomatic infected subjects (Cho et al., 2018). The SARS-CoV-2 infection occurs also through inhalation of oral droplets or by touching contaminated surfaces and then scrubbing nose, mouth, or eyes. Occasionally, the SARS-CoV-2 spread was documented by aerosols suspended in the air (Correia et al., 2020; Sabino-Silva et al., 2020). The SARS-CoV-2 incubation period ranges between 2 and 14 days (expected average around 5 days). The most frequent clinical outcomes include fever, cough, sore throat, headache, fatigue, myalgia, and breathlessness. Altogether, these initial symptoms are similar to other respiratory infections (Yang et al., 2020). The clinical features of COVID-19 ranges from asymptomatic state to death and also include self-limiting respiratory complications [acute respiratory distress syndrome (ARDS)] and severe progressive pneumonia (Cao et al., 2020; Chen et al., 2020; Huang et al., 2020).

Although the mortality rates of SARS-CoV-2 are debated, it is well established that elderly people (65 years and older) and subjects with other comorbidities such as cardiovascular diseases, hypertension, and diabetes mellitus (DM) are more susceptible to severe illness and subsequent mortality (Singh et al., 2020; Wang et al., 2020). Indeed, the prevalence of DM was the second most common comorbidity in several cohorts of COVID-19 patients (Stein, 2020; Stoian et al., 2020; Zhou F. et al., 2020). The DM prevalence is about 425 million worldwide, corresponding to 8.8% of adults between 20 and 79 years (Cho et al., 2018). It was suggested around 20% of a diabetic population without diagnostic in England and it is expected that this percentage is higher in lower-income countries (Clark et al., 2020). The association between DM and COVID-19 increases the risk of a more severe illness, ARDS, hospitalization, and death (Chan-Yeung and Xu, 2003). Multiple hypotheses have been proposed to support the association between DM and COVID-19 severity. Briefly, diabetic patients with inadequate metabolic control frequently present reduced anti-viral immune response, which is also associated with pathogen proliferation (Philips et al., 2003; Rothe et al., 2020). Moreover, DM is commonly accomplished by magnified reactive oxidative species (ROS) with no counter regulation by appropriate antioxidant defense, which can be further related with the exacerbated inflammatory response to SARS-CoV-2 and ARDS (Rothe et al., 2020).

Based on published evidence, we have focused on discussing the pathophysiological principles on infection and SARS-CoV-2 immune response of lung cells in normoglycemic and hyperglycemic conditions, and assessment findings to frame lung interactions between SARS-CoV-2 infection and DM, paving the way to better understand the unique characteristics of the SARS-CoV-2 infection in diabetic lungs. We also summarized current evidences of subcellular distribution of glucose transporters in lung and highlighted the effect of higher airway surface liquid (ASL) glucose concentration on SARS-CoV-2 proliferation, as well as its relationship with bacterial proliferation, inflammation, oxidative stress, and lung tissue injury.

### LUNG PHYSIOLOGY

The distal lung alveolar epithelium is mainly composed of two pneumocytes. Type I pneumocytes coat around 92% of lung alveolar surface and their vital function is to promote O_2_ and CO_2_ exchange through the alveolar-capillary barrier (Stone et al., 1992; Mossel et al., 2008). Type II pneumocytes secrete surfactant into the alveolar space, which is a biofluid capable to reduce the surface tension at the air/liquid interface (Veldhuizen and Haagsman, 2000) and hence also contribute to keeping the alveoli open and facilitate gas exchange. As expected, type II pneumocytes injury frequently reduces the surfactant secretion to the ASL in alveolar space, which is accomplished by reduction in lung compliance and atelectasis (Mossel et al., 2008). The maintenance of an adequate level of ASL with optimal levels of surfactant and balanced oxidative/antioxidative condition are pivotal to perform adequate lung function (Mossel et al., 2008).

In physiological conditions, the pneumocytes and pericytes contribute to provide a compartment barrier and vascular integrity. This alveolar-capillary barrier restrains the physical interactions between pneumocytes and immune cells, which is paramount to avoid inflammation. Additionally, it prevents coagulation due to the physiological secretion of coagulation inhibitors, glycoproteins, and glycolipids promoting a protective coat with anti-coagulation activity. In physiological circumstances, the desquamated alveolar cells display immunomodulatory responses to induce cytokine production, leukocyte recruitment, and scavenger properties, specially related with MHC class II-mediated antigen processing, which reinforces the role in immune surveillance against viruses and bacteria in the lung (Teuwen et al., 2020).

Angiotensin-converting enzyme 2 (ACE2) is one of the key players of the renin angiotensin aldosterone system (Jia et al., 2009). ACE2 is a classical type 1 integral membrane glycoprotein expressed by lung epithelial cells (Fang et al., 2020; Pal and Bhansali, 2020). In more detail, ACE2 expression has been detected in type I and type II pneumocytes in humans and animal models (Hamming et al., 2004; van den Brand et al., 2008); however, the ACE2 expression in type II is higher than in type I pneumocytes (Hamming et al., 2004; van den Brand et al., 2008). ACE2 hydrolyzes angiotensin II into Ang (1-7), which plays important anti-inflammatory and antioxidant roles to protect lungs against ARDS (Tikellis and Thomas, 2012; Zou et al., 2014). It is important to point out that ACE2 is also expressed in other lung immune cell as T and B lymphocytes, fibroblasts, natural killer (NK) cells, and monocytes (Cheng et al., 2020; Fu et al., 2020).

### Glucose Fluxes and Subcellular Distribution of Glucose Transporters in Lung

The regulation of ASL composition plays a pivotal role in lung defense (Baker et al., 2006b). Due to a counterbalance of glucose efflux and influx in the alveolar epithelia, the glucose concentration in ASL is around 0.4 mM, which correspond about 12 times lower than the plasma and extracellular liquid (ECL) (Baker et al., 2007; Baker and Baines, 2018). The fenestrated capillaries of the lung warrant passive communication of glucose between the blood and ECL. Despite the presence of tight junctions in alveolar epithelial cells, glucose molecules are capable to move from the ECL to the ASL through a paracellular pathway (Saumon et al., 1996). Glucose can also access the ASL via transcellular pathway when the intracellular glucose concentration is higher than ASL (Pezzulo et al., 2011). Bearing in mind that glucose can be transported by two types of glucose transporters, the Na(+)/glucose cotransporters (SGLT) and the facilitative glucose transporters (GLUT) (Sabino-Silva et al., 2010), it established that glucose uptake is generated by GLUT2 in proximal airways and through the SGLT1 in distal alveolar cells, leading to lower glucose concentration in ASL (Garnett et al., 2012b; Oliveira et al., 2016).

**FIGURE 1:**
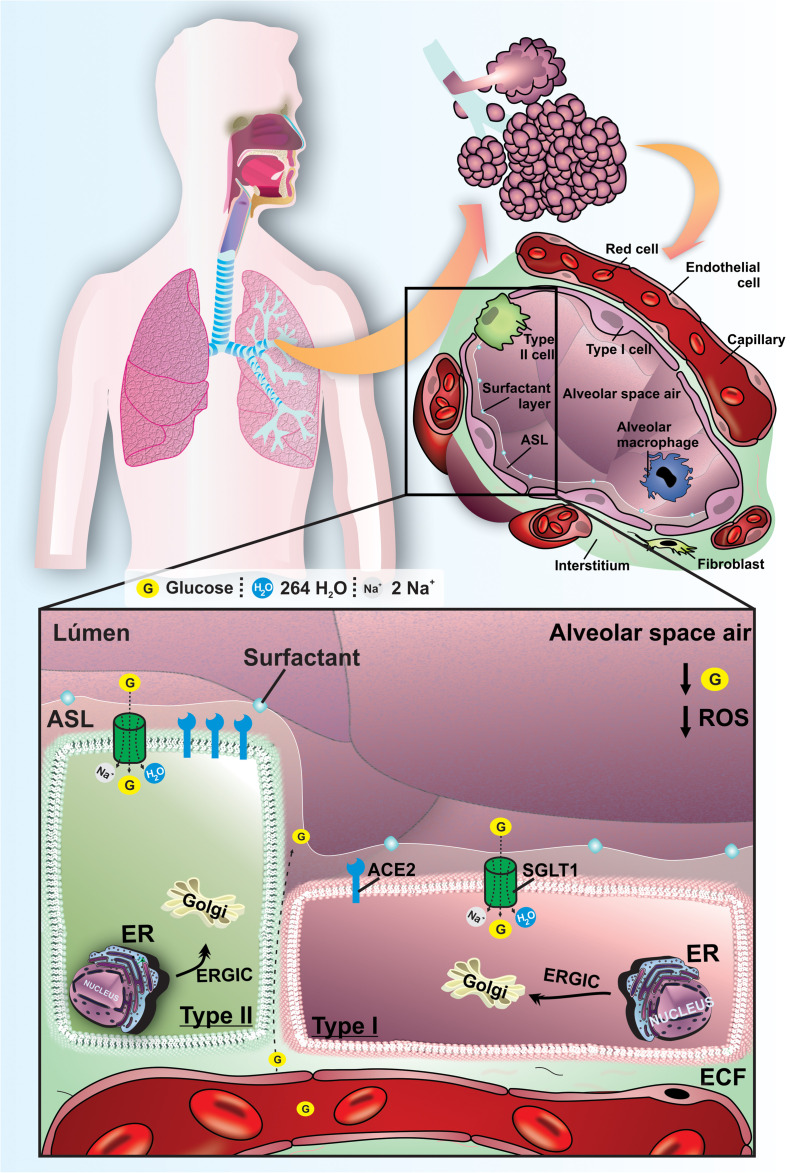
Functional mechanisms to maintain lung homeostasis and to regulated ASL glucose concentration in ASL of healthy normoglycemic subjects. Schematic representation of the lungs of normoglycemic subjects. The low concentration of glucose and the reduced volume in the ASL are mainly maintained by SGLT1 in the apical membrane of type I and II pneumocytes. ACE is present in pneumocytes, with greater expression in type II pneumocytes. The oxidative profile is in balance with low ROS production. G, glucose; SGLT1, Type 1 Na^+^/glucose/H_2_O cotransporter; ECF, extracellular fluid; ER, endoplasmic reticulum; ERGIC, endoplasmic reticulum-Golgi intermediate compartment; ACE2, angiotensin-converting enzyme 2; ROS, reactive oxygen species. ECL, extracellular liquid.

The glucose transport by GLUT2 and GLUT10 appears to be related with glucose regulation in lung proximal airways (Kalsi et al., 2009; Pezzulo et al., 2011; Garnett et al., 2012a). On the other side, the SGLT1 protein has been detected in both type I and type II pneumocytes (Bodega et al., 2010; Oliveira et al., 2016). The SGLT1 cotransport 2 Na^+^ ions, one molecule of glucose and 264 H_2_O in luminal membrane of pneumocytes I, and this protein can transport glucose into the cytoplasm of pneumocytes against its concentration gradient (Sabino-Silva et al., 2010). In fact, we have previously demonstrated the functional role of SGLT1 in distal alveolar cells by instillation of an SGLT inhibitor, which promoted an increase of ASL glucose concentration in non-diabetic animals and reinforced the pivotal role of SGLT1 to maintain low ASL glucose concentration. The pharmacological blockage of SGLT1 was also suitable to increase the ASL volume (Oliveira et al., 2016). Furthermore, it is established that Aquaporins (AQPs) also promotes water reabsorption in alveolar cells, which is paramount to maintain an adequate volume of ASL in alveolar space (Schmidt et al., 2017; Figure 1).

## Pathophysiology of DM in Lung

### Regulation of ASL Glucose Concentration in DM

Glucose diffuses mainly from plasma to ASL through paracellular pathway due to the glucose concentration gradient. DM is characterized by classical pre-existing vascular dysfunction. In this context, moderate injury in this epithelial barrier can occur in non-controlled diabetic patients (Li et al., 2019; Teuwen et al., 2020). As described in a normoglycemic condition, the ASL glucose concentration is also correlated with glycemia in DM (Baker et al., 2006b; Oliveira et al., 2016). In a hyperglycemic scenario, the referred increase in glucose flux from plasma to ASL is not counterbalanced by a parallel adjustment by SGLT1-mediated re-uptake of glucose, which explain the higher ASL glucose concentration in distal lung (Oliveira et al., 2016). Bearing in mind the osmotic effect due to the high ASL glucose concentration, these changes could explain the higher total water content in lung during hyperglycemic condition, which can be related with higher volume of ASL, leading to reduced gas exchange rates of O_2_ and CO_2_ between alveolar space and lung capillaries. Frequently, the mild increase in ASL can be compensated by ventilatory response mechanisms (Oliveira et al., 2016). It is important to point out that the pathological reduction of O_2_ and CO_2_ exchange through the lumen of pneumocytes and blood occurs in poorly controlled diabetic patients mainly under bacterial, inflammatory, or oxidant disruption (Stone et al., 1992; Mossel et al., 2008).

### Oxidative Stress and Inflammatory Outcomes of DM Related With ASL Glucose Regulation in Lung

Although ACE2 expression was described in type I and type II pneumocytes (Helenius and Aebi, 2001; Vrhovac et al., 2015), a distinguishing analysis on these cells was not shown in diabetic conditions. It revealed an increase in ACE2/ACE ratio in the lungs of long-term diabetics (Roca-Ho et al., 2017), indicating a shift to trigger inflammatory and ROS activities. The imbalance associated with higher ROS formation and reduced capability to detoxify the reactive intermediates promotes oxidative stress. It is currently accepted that hyperglycemia activates a metabolic signaling route, which culminates in higher levels of ROS formation (Volpe et al., 2018). Besides, several transcription factors related to ROS also trigger inflammation (Chatterjee, 2016). Additionally, inflammation leads immune cells to release cytokines to recruit additional immune cells to the oxidative stress region. Reflexively, higher levels of ROS delivery by immune cells also promotes tissue injury, triggering more inflammation (Chatterjee, 2016). We have also shown that oxidative stress dysregulation was parallelly associated with hyperglycemia and diabetic complications (Diniz Vilela et al., 2016) due to impairment of lipids and proteins in several tissues, such as lung (Baynes, 1991).

We also have previously demonstrated the increase of ASL glucose concentration promoted by SGLT1 inhibitors in the lungs of diabetic animals (Oliveira et al., 2016); however, the opposite effect on ASL glucose concentration was observed under the beta-adrenergic agonist application due to the SGLT1 translocation to plasma membrane of type I and type II pneumocytes. In this context, we also showed that SGLT1 inhibitors promote bronchial inflammation (interferon-γ and Interleucin-1β), reduction on antioxidant system, and atelectasis associated with significant decrease in survival rate in an ARDS promoted by cecum ligation and puncture (CLP)-induced sepsis animal model (Cardoso-Sousa et al., 2019). Otherwise, a specific β2-adrenergic agonist reduced bronchial inflammation (interleucin-1β), preserved higher levels of antioxidant system, and reduced pulmonary atelectasis associated with bronchodilation (Cardoso-Sousa et al., 2019). The SGLT1-triggered water transport is feasible directly together with classical Na^+^ and glucose transport, and also indirectly as a powerful facilitator of passive water cotransport (Erokhova et al., 2016). Accordingly, we suggested that SGLT1 translocation to plasma membrane of type I and type II pneumocytes could decrease the ASL volume (Oliveira et al., 2016), facilitating the O_2_ and CO_2_ exchange, which indirectly could reduce lung inflammation. Altogether, changes in lung SGLT1 translocation were linked with ASL glucose concentration, ASL volume, bronchial inflammation, antioxidant system, and atelectasis in diabetic models.

### Effect of DM on Lung Bacterial Proliferation Related With ASL Glucose Regulation

**FIGURE 2:**
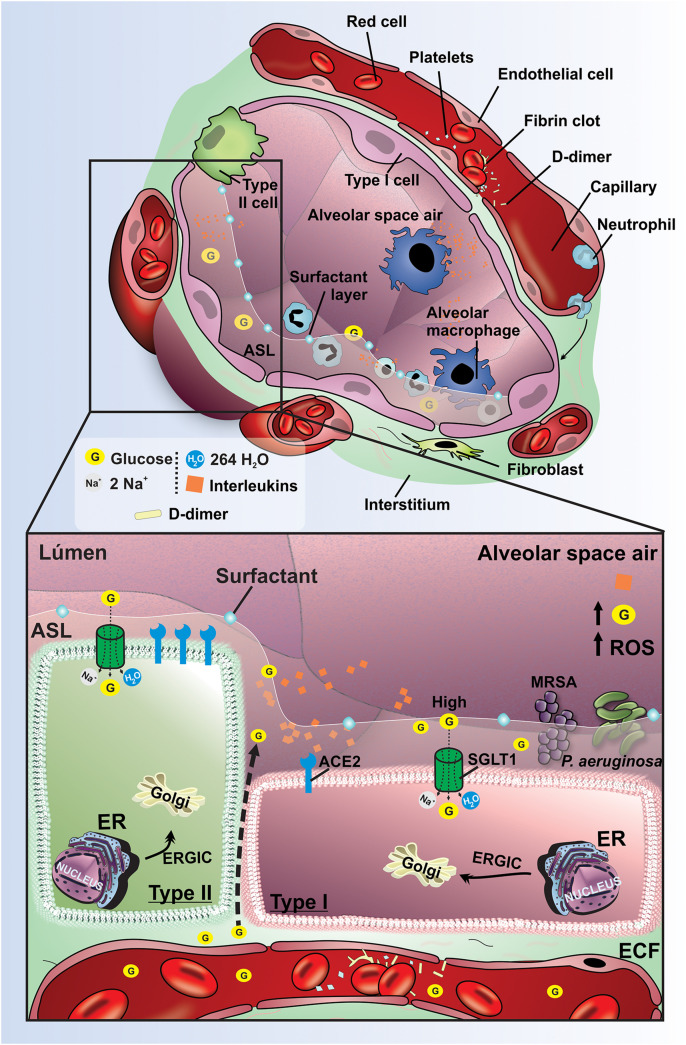
Pathophysiological mechanisms to regulate lung function and ASL glucose concentration effects on diabetic hyperglycemic patients. Schematic representation of pathophysiological mechanisms related to DM in the lung. The high concentration of glucose and the increased volume in the ASL are maintained mainly by the low expression of SGLT1 in the apical membrane of type I and II pneumocytes in the lungs of diabetics. Hyperglycemia promotes increased paracellular glucose transport from blood to ASL. The evolution of DM promotes activation of the inflammatory cascade with the production of interleukins, increased ROS, and endothelial damage. These changes provide an increased risk for pneumonia due to the proliferation of bacteria *P. aeruginosa* and MRSA. G, glucose; SGLT1, Na^+^/glucose/H_2_O type 1 cotransporter; ECF, extracellular fluid; ER, endoplasmic reticulum; ERGIC, endoplasmic reticulum-Golgi intermediate compartment; ACE2, receptor angiotensin-converting enzyme 2; ROS, reactive oxygen species; *P. aeruginosa*, *pseudomonas aeruginosa*; MRSA, Methicillin-resistant *Staphylococcus aureus*; Orange rhombus: interleukins.

Despite consistent airway exposure to bacteria, ASL is usually sterile (Pezzulo et al., 2011). The proportion between bacterial growth and bacterial killing in ASL guide the outcome to infection or sterility. Several innate immune mechanisms are activated to remove bacteria as antimicrobials, cough, phagocytes, and mucociliary clearance (Pezzulo et al., 2011). It is well recognized that the reduced ASL glucose concentrations is paramount to airway defense against infection, which is capable of restricting bacterial growth by the reduction in nutrient availability (Baker and Baines, 2018) and reduced immune mechanism (Pezzulo et al., 2011). In fact, the hyperglycemia and higher ASL glucose concentration in diabetic patients has been strongly related with higher prevalence of respiratory complications (Pezzulo et al., 2011) and also predisposes to bacterial respiratory infection (Garnett et al., 2013; Oliveira et al., 2016). Multiple respiratory pathogens such as methicillin-resistant *Staphylococcus aureus* (MRSA) and *Pseudomonas aeruginosa* are isolated more frequently from respiratory secretions of hyperglycemic patients, and it has been associated with increased glucose concentration in ASL (Philips et al., 2005; Baker et al., 2006a). Despite the direct effect of hyperglycemia on ASL glucose concentration, and consequently on bacterial proliferation (Baker et al., 2006b, 2007; Baker and Baines, 2018), we demonstrated the power of the SGLTs to modulate these effects in diabetic conditions (Oliveira et al., 2016; Figure 2). Indeed, we proved that the blockage of the SGLT1 transport in distal alveolar cells by instillation of phlorizin was also capable of promoting bacterial proliferation of MRSA and *P. aeruginosa* in both non-diabetic and diabetic condition (Oliveira et al., 2016), which support the relationship between high ASL glucose concentration and bacterial proliferation. Altogether, these *in vivo* studies appear to be in line with clinical outcomes in respiratory system of diabetic patients (Baker and Baines, 2018). The increased surveillance for an optimal glucose control may permit decreased numbers of hospitalizations and improve outcomes of diabetic patients (Shubrook et al., 2017).

Despite several clinical outcomes described in the respiratory system of diabetic subjects, the relationship of the currently most prominent endocrine disease and lung has been considered neglected (Khateeb et al., 2019). Dual SGLT1/SGLT2 inhibitors were considered in the DM treatment (Inagaki et al., 2013; Polidori et al., 2013; Powell et al., 2013); however, the definitive long-term adverse effects on respiratory tract especially in cases of associated bacterial and viral infections could be further studied. The component and volume regulations of ASL open perspectives to new treatments capable to reduce ASL glucose concentration associated with reduction of ASL volume. Additionally, it is important to address studies to evaluate the potential long-term effects of β-adrenoreceptor agonists in the lung due to the induced tolerance after prolonged administration (Raffay et al., 2014).

## Pathophysiology of COVID-19 in Lung

### The Replicative Cycle of SARS-CoV-2 in Alveolar Lung Cells

Severe acute respiratory syndrome coronavirus 2 is a positive single-stranded RNA (ssRNA [+]) virus, shielded by a nucleocapsid protein (N) shell and encompassed by a lipid bilayer conferring it a pleomorphic spherical shape. The envelope (E) and membrane (M) proteins and the spike (S) glycoproteins are anchored on its surface (Ding and Liang, 2020; Santos et al., 2020).

Briefly, the entry mechanism of SARS-CoV-2 into the host cells is similar to what occurs in the alveolar lung cells. The SARS-CoV-2 entry in alveolar cells includes receptor binding, proteolytic activation for membranes fusion, and viral internalization. The viral entry is promoted by interaction of S protein with the ACE2 in alveolar cells. The S protein is constituted by the S1 subunit (contain the receptor binding domain, RBD, which is responsible for interacting with host cell receptors) and the S2 subunit (triggers the fusion of viral and endosome membranes allowing viral entry into the host cells). The RBD/ACE2 interaction triggers the endocytosis of viral particle and endosome formation (Hoffmann et al., 2020a; Santos et al., 2020; Walls et al., 2020).

The host surface cell receptor ACE2 was recently recognized as a functional receptor of SARS-CoV-2 (Li et al., 2003). The ACE2 ectodomain possesses peptide motifs suitable to cleave several peptides. The activity of ACE2 can be managed by the attachment of ligand with ectodomain, receptor internalization, and transcription/translating interplay (Imai et al., 2005; Jia et al., 2009). In addition, since the entry of SARS-CoV-2 occurs connected with ACE2, it might decrease the functional ACE2 in the lung (Lukassen et al., 2020). Later, a cleavage of the S1/S2 subunits by host proteases, such as furin and transmembrane protease/serine (TMPRSS), exposes the S2 subunit enabling the fusion of SARS-CoV-2 and endosome membranes. These proteases are attached on the cellular surface, among them, the TMPRSS2 was described as an important protease for SARS-CoV-2 entry in lung cells (Lukassen et al., 2020). To support the crucial role of molecules derived from glucose and carbohydrates in the attachment with receptors, the N-linked glycans adhered in S protein can also modulate the effect of activated proteases (Walls et al., 2016).

The N-terminal domain (NTD) on S1 subunit of coronaviruses is suitable to bind in carbohydrate moieties (sugar-binding galectin motifs and 9-O-acetylated neuraminic acid) (Peng et al., 2011), while the C-domain (CTD) is capable to connect to protein receptors as ACE2. The S1 domains are extensively associated with N-linked glycans, which are pivotal to virion attachment (Helenius and Aebi, 2001). Recently, it was suggested that carbohydrates derived from glucose molecules, as the 22 N-linked glycosylation sequons, and other oligosaccharides are attached into S protein to aid the interaction between SARS-CoV-2 and host cell receptors (Grant et al., 2020), suggesting a potential effect of a hyperglycemic milieu. A significant part of S protein is covered by glycans; however, the ACE2 binding domain in the S1 subunit is uncovered by glycans, which can be paramount to SARS-CoV-2 entry in host cells (Watanabe et al., 2020) and open perspectives to new therapeutic strategies.

**FIGURE 3:**
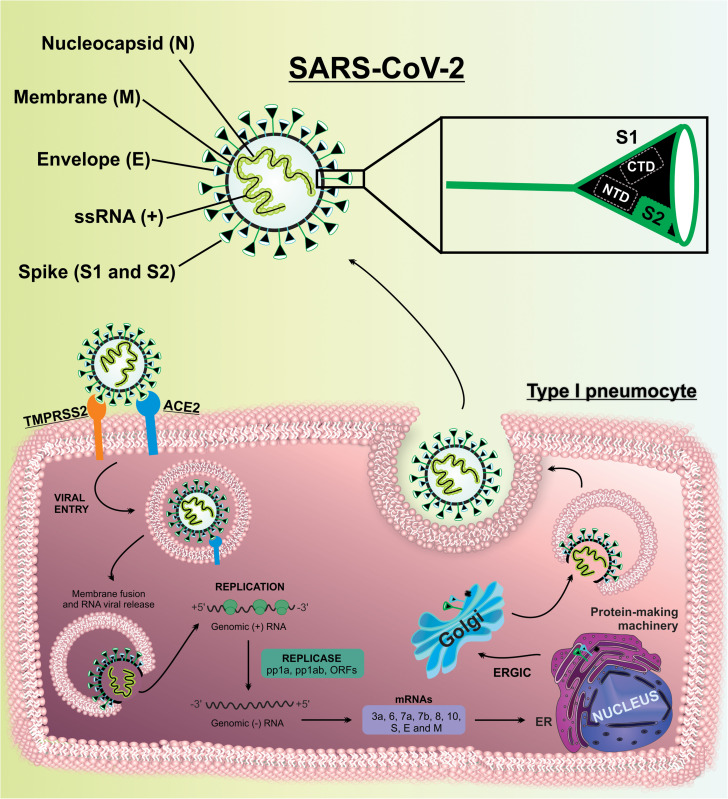
SARS-CoV-2 structure and SARS-CoV-2 replication cycle in lung cells. The main structural proteins of SARS-CoV-2 are spike (S), envelope (E), membrane (M), and nucleocapsid (N) proteins. The S, E, and M proteins are assembled in the viral membrane and the N protein merge in the viral RNA. SARS-CoV-2 interacts with ACE2, and subsequently with TMPRSS2, in lung cells. After SARS-CoV-2 entry occurs the release of viral genome and replication. Structural and non-structural proteins are assembly and packaging to SARS-CoV-2 release by exocytosis.

After SARS-CoV-2 viral entry occurs, the fusion between the viral envelope with endosome membrane, resulting in the exposure of the nucleocapsid. Subsequently, the viral uncoating is triggered and the viral genome is released into the cytoplasm. The exposure of the RNA permits the viral replication, and genomic RNA is partially translated to produce non-structural proteins (nsps) from two open reading frames (ORFs), ORF1a and ORF1b. The ORF1a yield polyprotein 1a (pp1a) is subsequently cleaved into 11 nsps. The continued translation of ORF1b produces the polyprotein 1ab (pp1ab), which is cleaved into 15 nsps. Proteolytic cleavages are mediated by viral proteases nsp3 and nsp5 (Belouzard et al., 2009; Kim et al., 2020). Nsps will assemble to form a replicase-transcriptase complex (RTC), which is responsible for RNA synthesis, replication, and transcription of nine subgenomic RNAs (sgRNAs) (Fehr and Perlman, 2015). The viral genomic RNA is transcribed into negative-strand RNAs, which are intermediates of genome replication by serving as a template for the synthesis of positive-sense genomic RNAs and sgRNAs (Kim et al., 2020). The sgRNAs act as mRNAs for structural and accessory genes localized downstream of the replicase polyproteins (Sethna et al., 1991; Fehr and Perlman, 2015). SARS-CoV-2 is known to have 6 accessory proteins (3a, 6, 7a, 7b, 8, and 10) (Kim et al., 2020). The structural proteins S, E, and M are translated from short sgRNAs, inserted in the endoplasmic reticulum (ER), and directed to an intermediate compartment of ER with Golgi (ERGIC). Viral genomes are encapsulated by N protein and assembled with the structural proteins to form virus particles (Siu et al., 2008; Fehr and Perlman, 2015), where the M protein binds to E protein and later to the nucleocapsid. Finally, the S protein is incorporated into virions, completing the virion assembly, and then transported to the cell surface in vesicles, and released by exocytosis (Ye and Hogue, 2007; Kuo and Masters, 2013; Fehr and Perlman, 2015; Bojkova et al., 2020; Zhou Y. et al., 2020; Figure 3).

### Clinical Course of COVID-19

It is important bear in mind the clinical characteristics of COVID-19 to understand specific phenotype factors and evaluate the immunomodulation response in normoglycemic and hyperglycemic conditions. In this context, a three phase clinical classification to COVID-19 was proposed: (1) viremia phase; (2) acute pulmonary phase; (3) severe hyperinflammation phase (Cao and Li, 2020; Siddiqi and Mehra, 2020).

(1) *Viremia phase*: The incubation period occurs in early stages of infection of SARS-CoV-2 and is characterized by the viral entry into host cells, replication and establishment of infection mainly in the respiratory system. After the incubation period, the viremia phase can be asymptomatic or related to mild symptoms (Cao and Li, 2020; Siddiqi and Mehra, 2020). In this phase, the diagnostic is frequently performed by RT-PCR using nasopharyngeal swabs samples or, more recently, saliva (Sabino-Silva et al., 2020). The probability of SARS-CoV-2 detection increases with multiplication of SARS-CoV-2 and the presence of lymphopenia and neutrophilia is suitable. It also reported changes in high resolution computer tomography (HRCT) of the chest and presence of IgM against SARS-CoV-2. The seroconversion can occur 4 days after symptoms onset; however, it occurs in high frequency after 14 days. It is expected that immunocompetent subjects with additional risk factors are capable of generating sufficient immune responses to strongly suppress the SARS-CoV-2 replication (Cao and Li, 2020; Sethuraman et al., 2020; Siddiqi and Mehra, 2020).
(2) *Acute pulmonary phase*: In this period, the presence of several symptoms is expected as cough, fever, and occasionally shortness of breath. It is characterized as mild symptoms in the absence of hypoxia (PaO2/FiO2 > 300 mmHg), and severe under hypoxia (PaO2/FiO2 < 300 mmHg). HRCT of the chest can indicate lung infiltrates and typical COVID-19-ground-glass opacities. The lymphopenia and systemic inflammatory biomarkers can be higher in blood tests. As expected, the changes in chest imaging and blood test parameters are in accordance with severity degree (Siddiqi and Mehra, 2020). Most patients in this phase are also capable of suppressing SARS-CoV-2 infection with no exacerbated immunomodulatory response.
(3) *Severe hyperinflammation phase*: A minority of SARS-CoV-2 infected patients develop the most severe phase of the disease, which is characterized as pulmonary and systemic hyperinflammation due to the elevation of several inflammatory markers as IL-2, IL-6, IL-7, D-dimer, C-reactive protein, tumor necrosis factor-α, macrophage inflammatory protein 1-α, and ferritin (Siddiqi and Mehra, 2020). The characteristics in this phase can be similar to ARDS, which determines the presence of non-cardiogenic pulmonary edema, hypoxia, and, frequently, mechanical ventilation demand (Matthay et al., 2018). To note, the hypoxic respiratory failure due to ARDS is the leading mortality cause in SARS-CoV-2 infected patients. Additionally, the ARDS has been related to damages in the integrity of the alveolar-capillary barrier, which mediate additional inflammatory cell infiltration and development of pro-coagulative markers.

Usually, COVID-19 patients in severe hyperinflammation display lung vascular leakage and airway liquid accumulation (pulmonary edema) as a result of multiple mechanisms. Considering the ability of SARS-CoV-2 to gain entry in type I and type II pneumocytes and also in vasculature endothelial blood vessels cells by the expression of ACE2, it is expected to have a diffuse inflammatory profile in these epithelial cells that can lead to cellular lysis and apoptosis. The cell death in this region permits the fluxes of a protein-rich exudate biofluid derived from plasma and extracellular fluid to the alveolar space. As expected, the presence of this rich-protein biofluid in alveolar space can reduce the O_2_ and CO_2_ diffusion due to the increase of liquid layer and presence of proteins, which lead to higher fluid density. In moderate and severe fluxes of this protein-rich exudate biofluid, the compensatory ventilatory response can be inefficient to maintain adequate exudate O_2_ and CO_2_ exchanges (Oliveira et al., 2016). Considering the resemblance between inflammatory response in the lung of patients with COVID-19 and sepsis (Tomar et al., 2020), it is expected that SGLT1 is also reduced in luminal membrane of pneumocytes (Cardoso-Sousa et al., 2019), which can resonate in ASL glucose concentration and ASL volume repercussion. Additional effort needs to be expended to raise this question.

**FIGURE 4:**
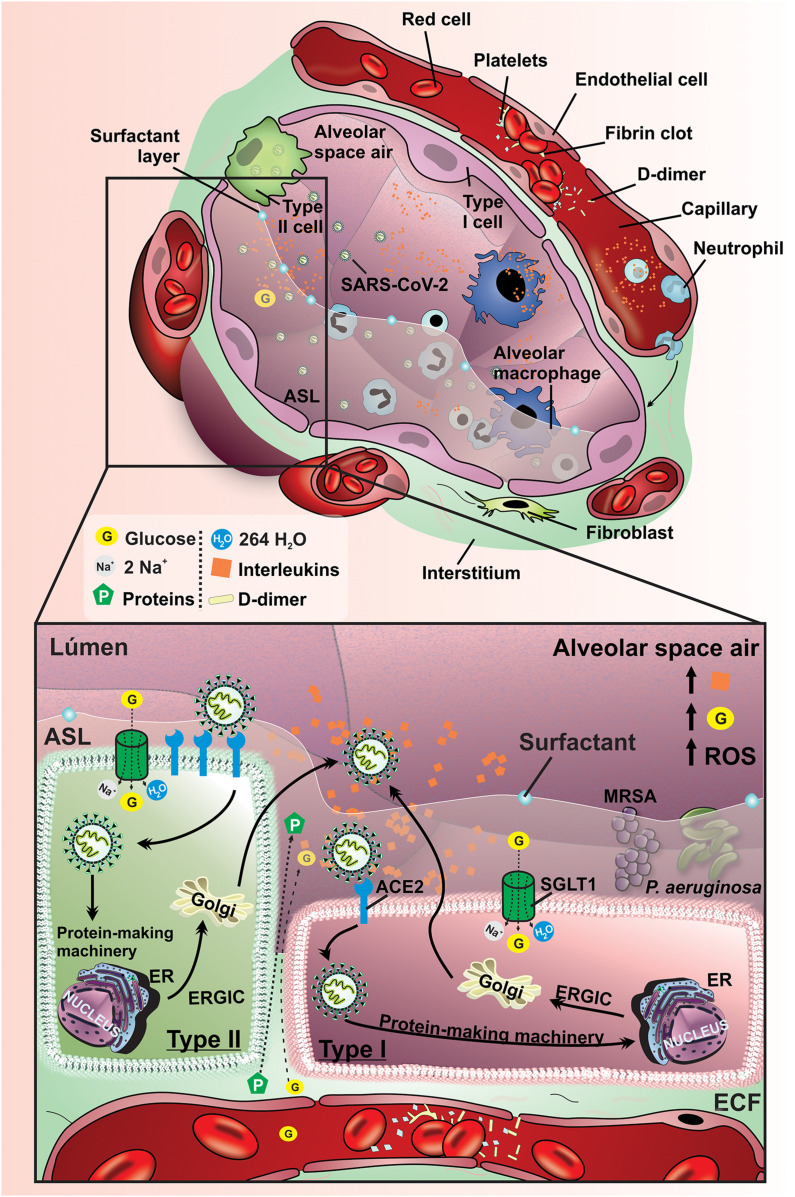
Pathophysiological mechanisms affecting lung function and ASL glucose concentration COVID-19 patients. Schematic representation of pathophysiological mechanisms related to SARS-CoV-2 infection in the lung of non-diabetic subjects. Spike glycoproteins from the SARS-CoV-2 envelope binds to ACE2, allowing the inoculation of the viral genome into type I and II pneumocytes. The virus uses the host cells machinery to replicate and infect other cells, which can result in the activation of an inflammatory cascade as well as promoting damage to the vascular endothelium. These changes promote an increase of the production of interleukins, activation of ROS, damage to the alveolar epithelium and endothelium, which allows the leakage of liquid and glucose from the interstitium into the alveoli. These changes provide an increased risk for pneumonia due to the proliferation of bacteria *P. aeruginosa* and MRSA. G, glucose; SGLT1, Na^+^/glucose/H_2_O type 1 cotransporter; ECF, extracellular fluid; ER, endoplasmic reticulum; ERGIC, endoplasmic reticulum-Golgi intermediate compartment; ACE2r, receptor angiotensin-converting enzyme 2; ROS, reactive oxygen species; *P. aeruginosa*, *Pseudomonas aeruginosa*; MRSA, Methicillin-resistant *Staphylococcus aureus*; Orange rhombus: interleukins.

The decrease in the functional ACE2 in the lung can activate the kallikrein–bradykinin system by an indirect pathway, promoting increase in vascular permeability. Besides, it is capable of activating neutrophils and secreting ROS. Furthermore, immunoinflammatory cytokines can promote additional inter-endothelial gaps. The acid hyaluronic pathway is also activated by inflammatory cells leading to fluid retention in alveolar space. Altogether, these several pathophysiological and immunological mechanisms stimulate vascular leakage and increased vascular permeability leading to pulmonary symptomatology in COVID-19 patients (Figure 4).

## Pathophysiology of COVID-19 in the Lung of Diabetic Patients

### Effects of SARS-CoV-2 on the Lung of Diabetic Patients

The general mechanism of SARS-CoV-2 infection in hyperglycemic diabetic subjects is similar to normoglycemic subjects; however, we point out the potential pathophysiological changes in the lung of diabetic patients suitable to changing the clinical course of COVID-19. In a genome-wide association, type I and type II DM were also related with higher ACE2 expression in lung study based on genotype expression of 5,515 subjects (Rao et al., 2020). Until now, the prevalence of a diabetic population with COVID-19 is similar with DM prevalence among general population, which indicates similar susceptibility to SARS-CoV-2 infection (Apicella et al., 2020; Jeong et al., 2020). In a pathophysiological context, it suggests similar ACE2 expression in nasal and oral mucosal cells in both normoglycemic and hyperglycemic populations. On the other hand, the increase of ACE2 in type I and type II pneumocytes (Roca-Ho et al., 2017) can be related with parallel changes in severity of COVID-19 in diabetic comparing to normoglycemic population, which is also reflected in higher mortality in diabetic subjects (Jeong et al., 2020). Thus, the higher ACE2 expression in pneumocytes could promote parallel entry and replication of SARS-CoV-2, which can be associated with the severity of COVID-19 in diabetics.

The higher risk to develop worse outcomes in diabetic population infected with SARS-CoV-2 (Apicella et al., 2020) also can be associated with the reduced ACE2/ACE ratio in diabetic condition, which could be critical to several pathophysiological mechanisms of COVID-19 (Roca-Ho et al., 2017). To counter regulate the opposite effects of ACE, it is important to emphasize that ACE2 expression is pivotal to promote anti-inflammatory, antioxidative stress and antifibrotic adjustments in the lung and to keep the vascular integrity of lung capillaries (Dalan et al., 2020). However, the reduced ACE2/ACE ratio is a classical characteristic that could be associated evaluating anti-inflammatory and antioxidant systems, which is reduced in the lung of diabetic mice (Roca-Ho et al., 2017).

In the second step, in the SARS-CoV-2 entry also occurs the internalization of ACE2, which can reduce the probability of additional virions entry in these infected cells. Dichotomously, it promotes additional pro-inflammatory and pro-fibrotic regulations in the lung, facilitating microvascular leakage in the respiratory system due to ACE-Ang-II-AT1R axis, since the decrease in ACE2 will decrease the Ang 1-7 formation, which has anti-inflammatory effects (Dalan et al., 2020). This evidence suggests a worst clinical scenario in DM including (i) higher ACE2 expression facilitating the SARS-CoV-2 infection, and (ii) a reduced ACE2/ACE ratio post SARS-CoV-2 infection triggering an heightened inflammatory and oxidative responses in diabetic patients increase the risk of a more severe form of COVID-19, especially in the acute pulmonary and severe hyperinflammation phase (Volpe et al., 2018).

Recently, it was described that glucose concentration could be directly related with higher levels of SARS-CoV-2 in monocytes from bronchoalveolar lavage (BAL), which was related with glycolysis to produce ATP and with a new metabolic and proteomic profile triggered by glucose (Codo et al., 2020). It is already established that beta-coronaviruses, as SARS-CoV-2, need the cellular machinery for viral replication (Walls et al., 2020). The inhibition of SARS-CoV-2 replication due to reduced glucose transport by 2-deoxy-D-glucose (2-DG) and the ATP synthase inhibition by oligomycin suggests that the glucose metabolism in lung cells can develop a pivotal role in COVID-19 (Codo et al., 2020). Besides, higher glucose levels were also associated with parallel expression of IL-1β and other proinflammatory cytokines as TNF-α, IL-6, and IFN-α, IFN-β, and IFN-λ (Codo et al., 2020). These cytokines play critical roles in the cytokine storm and lung injury of COVID-19 patients (Shi et al., 2020), because under high glucose conditions SARS-CoV-2 infected monocytes can promote pulmonary epithelial cell death (Codo et al., 2020). These changes in inflammatory profile in diabetic condition are identical to the profile described in severe COVID-19 patients with the worst clinical outcome (Lucas et al., 2020). It was demonstrated that an elevation in inflammatory cytokine levels in acute pulmonary phase was related with poor clinical outcomes. The inflammatory cytokines signature is similar in COVID-19 patients that develop moderate and severe outcomes during 10 days from symptom onset; subsequently, in moderate patients this inflammatory profile declined, and it remains maintained and elevated in severe patients (Lucas et al., 2020), suggesting parallel regulations in diabetes.

The higher viral replication in hyperglycemic condition (Codo et al., 2020) also produces rapid cell death by apoptosis, increasing the immune cells recruitment. Subsequently, an excessive alveolar exudative and interstitial inflammatory reaction might occur as outcome. In diabetes, the hypersecretion of the pro-inflammatory cytokines can provoke the “cytokine storm,” resulting in lung tissue destruction by cytotoxic granules in patients with the worst clinical outcomes. The alveolar cell destruction leads to additional recruitment of immune cells with an excessive alveolar exudative and interstitial inflammatory reaction (Mirastschijski et al., 2020).

Angiotensin-converting enzyme 2 expression in the lung is upregulated on SARS-CoV-2 infection, with type 2 pneumocytes potentially serving as a key cell type facilitating pulmonary inflammation (Drucker, 2020); notably, interleukin IL-6 may be further exaggerated in response to a stimulus as seen in diabetic patients with COVID-19 (Chan-Yeung and Xu, 2003). The additional expression of ACE2 in type II alveolar cells are capable to promote cell death after the SARS-CoV-2 entry (Hoffmann et al., 2020b; Zhou F. et al., 2020). In diabetes, the additional damage in type II alveolar cells infected by SARS-CoV-2 drastically reduces pulmonary surfactant production and secretion to the alveolar space causing atelectasis, reduced blood oxygenation, lung fibrosis, edema, impaired regeneration, and ultimately, leading to respiratory failure (Alcorn, 2017; Fang et al., 2020).

In this context, the SARS-CoV-2 infection in diabetic patients makes them more prone to develop severe stages of diseases, ARDS, and increased mortality (Wu and McGoogan, 2020). In summary, DM, promotes changes in immunity, increase glucose concentration, exudate, and fluid volume in the ASL, as well as endothelial lesion with formation of disseminated intravascular coagulation (Tang et al., 2020). Thus, SARS-CoV-2 infection in diabetic patients causes a potential reduction in gas exchange, hypoxemia, due to the damage in both ventilation and tissue perfusion. Moreover, the association between DM and SARS-CoV-2 increases the protein and glucose concentration in ASL, leading to higher risk of pulmonary infections (Gao et al., 2020).

### Clinical Course of COVID-19 Diabetic Patients

The clinical course of COVID-19 in diabetic patients is similar to the clinical course described previously to normoglycemic patients. However, the prevalence of more severe phases as acute pulmonary and severe hyperinflammation phases is more frequent (Hussain et al., 2020; Pal and Bhansali, 2020; Singh et al., 2020). The classical pathophysiology of ARDS is focused on fibrin-rich exudates triggers by activation of the coagulation system and inhibition of fibrinolysis. A subgroup of patients with COVID-19-associated ARDS with higher mortality is characterized by low pulmonary compliance and high plasmatic D-dimer concentration. The endothelial dysfunction in the lung is critical to reduce gas exchange and to induce pro-thrombotic responses, which is associated with the worst prognosis (Grasselli et al., 2020). In this context, the endothelial dysfunction related to diabetes is associated with pro-inflammatory, pro-thrombotic, and significantly increased intracellular ROS (de Jongh et al., 2004; Schnabel et al., 2013; Khalaf et al., 2020). Furthermore, diabetic patients with COVID-19 exhibit disseminated intra-vascular coagulation and higher levels of D-dimer, leading to more severe endothelial injury and coagulation abnormalities, which has been associated with a higher mortality rate (Shi et al., 2020; Zhu et al., 2020). Other research groups also showed that D-dimer levels are higher in diabetic patients (Bhandari et al., 2020) and it is in accordance with higher levels of D-dimer in COVID-19 patient when glucose level is higher than 11 mmol/L, which was associated with parallel death rate (Li et al., 2020). D-dimer was also increased in uncontrolled glycemic subjects compared to optimal controlled diabetic subjects (Bhandari et al., 2020), reinforcing the importance of adequate surveillance in glycemic control to COVID-19 patients. Therefore, scientific evidence shows that optimal glucose control in diabetic patients with COVID-19 was associated with a significant reduction of inflammatory cytokines and D-dimer levels, decreasing the risk of COVID-19 complications. Poorly controlled DM has been linked to inhibited lymphocyte proliferative response to different kinds of stimuli, as well as impaired monocyte/macrophage and neutrophil functions (Hussain et al., 2020). A recent study with 595 consecutive hospitalized COVID-19 patients demonstrates that pneumonia and secondary infection were 2 times higher in diabetic than normoglycemic patients, which was parallel with changes in death rate (Akbariqomi et al., 2020). Thus, the adequate diabetes treatment maintaining optimal glucose levels may be an effective method for achieving glycemic targets and reducing mortality in diabetic patients with COVID-19 (Sardu et al., 2020).

**FIGURE 5:**
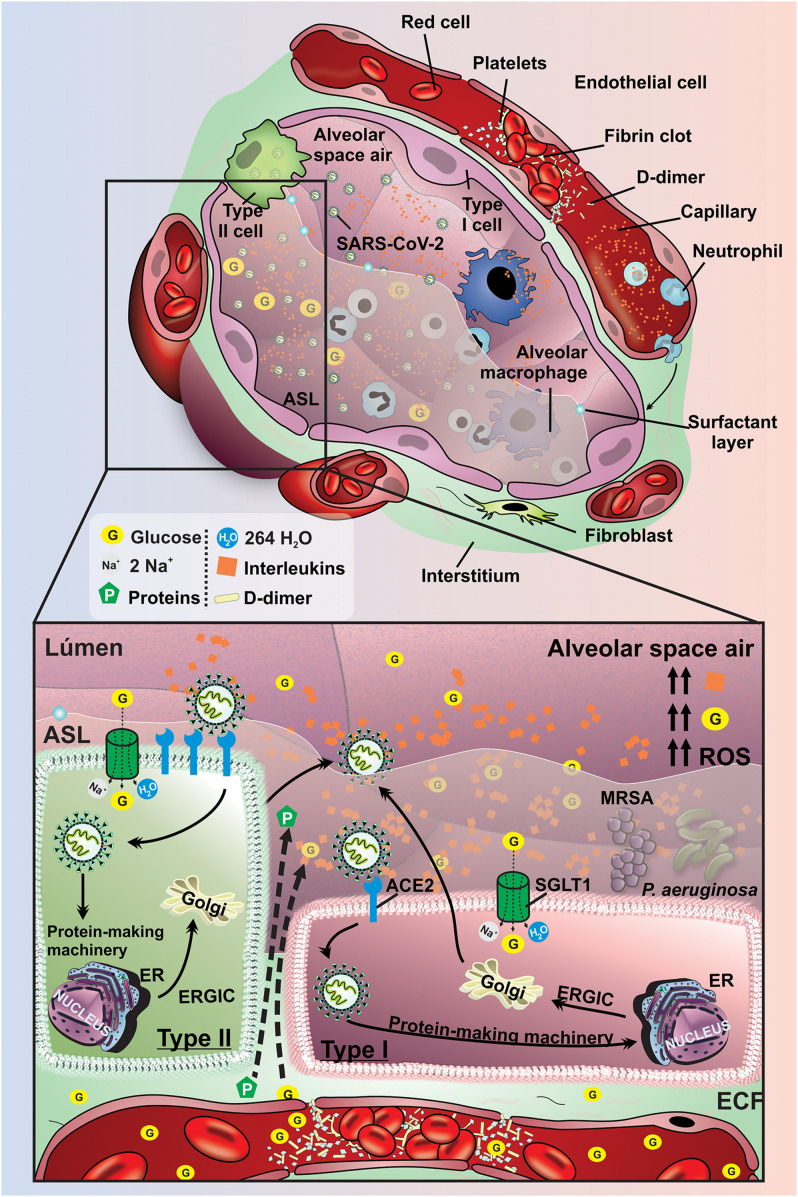
Pathological manifestations of lung tissue in diabetic patient affected by SARS-CoV-2. Schematic representation of pathophysiological mechanisms related to SARS-CoV-2 infection in the lung of diabetic subjects. Spike glycoproteins from the SARS-CoV-2 envelope binds to ACE2 allowing the inoculation of the viral genome into type I and II pneumocytes. The virus uses the host cells machinery to replicate and infect other cells which can result in the activation of a disseminated inflammatory cascade. The high concentration of glucose and the increased volume in the ASL are maintained mainly by the low expression of SGLT1 in the apical membrane of type I and II pneumocytes in the condition of DM. These changes promote a potential increase in the production of interleukins, activation of ROS, damage to the alveolar epithelium and endothelium, which allows the leakage of liquid and glucose from the interstitial to the alveoli. In addition, the risk of developing pneumonia due to the proliferation of *P. aeruginosa* and MRSA bacteria is greatly increased in diabetic individuals with COVID-19. G, glucose; SGLT1, Na^+^/glucose/H_2_O type 1 cotransporter; ECF, extracellular fluid; ER, endoplasmic reticulum; ERGIC, endoplasmic reticulum-golgi intermediate compartment; ACE2, receptor angiotensin-converting enzyme 2; ROS, reactive oxygen species; *P. aeruginosa*, *Pseudomonas aeruginosa*; MRSA, Methicillin-resistant *Staphylococcus aureus*; Orange rhombus: interleukins.

Taken together, it is pivotal to study the association between these synergic mechanisms to understand the effect of oxidative stress and inflammation in the lung of diabetic patients. Finally, studies have shown that patients with SARS-CoV-2 can develop secondary bacterial pneumonia and consequently worsen their clinical condition (Dong et al., 2020; Jin et al., 2020). SARS-CoV-2 can infect and kill alveolar pneumocytes and macrophages, leading to increased susceptibility to secondary bacterial pneumonia (Rothan and Byrareddy, 2020; Xu et al., 2020). Several pathophysiological mechanisms involved in worst clinical outcomes described in diabetes are summarized in Table 1. Thus, the risk of developing secondary bacterial pneumonia by dual SGLT inhibitors in diabetic patients with SARS-CoV-2 should be considered. On the other hand, the blocked SGLT1 in luminal membrane of type I and type II cells could be capable to reduce the intracellular glucose fluxes, which can reduce SARS-CoV-2 proliferation (Figure 5).

**TABLE 1:** Multifactorial mechanisms involved to the higher risk of worse outcomes in diabetic subjects associated with COVID-19.

Effectors in diabetic *compared* to normoglycemic condition	SARS-CoV-2 related mechanisms in diabetic compared to normoglycemic condition	Reference
↑ Expression of ACE2 in lung	↑ SARS-CoV-2 entry in respiratory cells	Roca-Ho et al., 2017; Rao et al., 2020
↓ ACE2/ACE ratio	↓ Anti-inflammatory, antifibrotic and antioxidant systems	Dalan et al., 2020
Internalization of ACE2 in the SARS-CoV-2 entry	↑ Pro-inflammatory, fibrotic and oxidant systems	Dalan et al., 2020
↑ Glucose concentration in bronchoalveolar lavage and ↑ Intracellular ATP production	↑ SARS-CoV-2 levels and ↑ levels of proinflammatory cytokines as IL-1β, TNF-α, IL-6, and IFN-α, IFN-β, and IFN-λ in lung	Codo et al., 2020
Inflammatory cytokines signature	Parallel inflammatory cytokine levels in COVID-19 patients with diabetes and COVID-19 patients with worst clinical outcomes	Codo et al., 2020; Lucas et al., 2020
↑ Viral replication in hyperglycemic condition	↑ Apoptosis, ↑ immune cells, ↑alveolar exudative and ↑interstitial inflammatory recruitment	Codo et al., 2020; Mirastschijski et al., 2020
Additional damages in type II alveolar ↑ viral replication in hyperglycemic condition	↓ Surfactant production causing atelectasis, reduced blood oxygenation, lung fibrosis and lung edema	Alcorn, 2017; Fang et al., 2020
↑ Endothelial dysfunction related to diabetes	Additional increase in ROS production, ↑ pro-inflammatory and ↑ Pro-thrombotic	de Jongh et al., 2004; Khalaf et al., 2020
↑ Protein and glucose concentration in ASL	↑ Risk of secondary infections	Oliveira et al., 2016; Gao et al., 2020;
↑ Levels of D-dimer	↑ Endothelial injury and coagulation abnormalities, which has been associated with a higher mortality rate	Shi et al., 2020; Zhu et al., 2020

Diabetes mellitus are definitely at an increased risk of severe COVID-19 outcomes. Hence, it is advisable that community-dwelling residents having underlying DM take extra precautions to not contract the virus by adopting social distancing and strict hand and respiratory hygiene (Chan-Yeung and Xu, 2003).

## Final Remarks

Altogether, the latest advances in pathophysiology have challenged the understanding about the lung environment under normoglycemic and hyperglycemic conditions. The functional regulation of glucose/water transporters in luminal membrane of type I and type II pneumocytes and the pivotal role of ASL glucose concentration modulation broaden the horizons in lung pathogenesis of diabetic patients infected with SARS-CoV-2. The global spread of COVID-19 has led to an urgent effort to draw the complex pathophysiology of the SARS-CoV-2 infections in the lung of diabetic patients. In this context, we described the current view about the effects of higher ASL glucose concentration, intracellular glucose metabolism, and glycans in the bacterial proliferation, inflammation, oxidative stress, and pro-thrombotic responses in the lung, which frequently leads to harmful clinical outcomes in diabetic patients. Finally, this draw of the lung interaction between SARS-CoV-2 infection and DM paves the way to better understand unique characteristics of the SARS-CoV-2 in lung of diabetic patients, indicating that advances in intensive glycemic control may be an effective method for reduce mortality in diabetic patients with COVID-19. In summary, DM promotes an increase in ASL glucose concentration, ASL volume accumulation in alveolar space, imbalance of ROS, and inflammatory chemokine production. The COVID-19 infection triggers type I and type II pneumocytes damages and additional lung endothelial lesions, with subsequent additional secretion of protein-rich inflammatory fluid in the alveolar space and intravascular coagulation in lung vessel, which leads to a reduction in surfactant and gas exchange. As expected, the prevalence and severity of hypoxemia, severe hyperinflammation, and pro-thrombotic responses and pneumonia are higher in COVID-19 diabetic patients.

## Author Contributions

All authors listed have made a substantial, direct, and intellectual contribution to the work and approved it for publication.

## Conflict of Interest

The authors declare that the research was conducted in the absence of any commercial or financial relationships that could be construed as a potential conflict of interest.
